# On the Influence of Manufacturing Parameters on the Microstructure, Mechanical Properties and Corrosion Resistance of AISI 316L Steel Deposited by Laser Engineered Net Shaping (LENS^®^)

**DOI:** 10.3390/ma16051965

**Published:** 2023-02-27

**Authors:** Magdalena Rzeszotarska, Dariusz Zasada, Tomasz Płociński, Wojciech J. Stępniowski, Marek Polański

**Affiliations:** 1 Department of Functional Materials and Hydrogen Technology, Institute of Materials Science and Engineering, Military University of Technology, Kaliskiego 2 St., 00-908 Warsaw, Poland; 2 Department of Structural Materials, Institute of Materials Science and Engineering, Military University of Technology, Kaliskiego 2 St., 00-908 Warsaw, Poland; 3 Faculty of Materials Science and Engineering, Warsaw University of Technology, Wołoska 141 St., 02-507 Warsaw, Poland

**Keywords:** additive manufacturing, LENS^®^, DED, 316 SS, parameters, corrosion resistance, mechanical properties

## Abstract

Samples of 316L SS were manufactured by Laser Engineered Net Shaping (LENS^®^) using different technological parameters. The deposited samples were investigated in terms of microstructure, mechanical properties, phase content and corrosion resistance (salt chamber and electrochemical corrosion). Parameters were chosen to obtain a proper sample built for layer thicknesses of 0.2, 0.4 and 0.7 mm by changing the laser feed rate while keeping the powder feed rate constant. After a comprehensive analysis of the results, it was found that the manufacturing parameters slightly affected the resulting microstructure and also had a minor impact (almost undetectable considering the uncertainty of the measurement) on the mechanical properties of samples. Decreases in resistance to electrochemical pitting corrosion and environmental corrosion with an increased feed rate and a decrease in layer thickness and grain size were observed; however, all additively manufactured samples were found to be less prone to corrosion than the reference material. In the investigated processing window, no influence of deposition parameters on the phase content of the final product was found—all the samples were found to possess austenitic microstructure with almost no detectable ferrite.

## 1. Introduction

At present, there is a significant increase in demand for modern structural materials. There has been a visible major advance in the design and production of these materials, as well as the growing importance of the quality of manufactured parts and machines. Steels are still the most common structural materials. Many hundred types of steel are currently manufactured, each belonging to a special group with specific characteristics and potential applications in industries such as energy, transport, construction and medicine. Despite many years of applications, they are still prospective materials, and new alloys are still being developed since they are usually characterised by relatively low prices in relation to other structural materials with good or very good mechanical properties. Austenitic stainless steels are very commonly used in different applications, from household machinery to medicine. They are very resistant to many types of corrosion and remain paramagnetic despite their high iron content. Currently, there are well-known and effective methods for the manufacturing of stainless steel parts, such as machining, powder metallurgy and different methods of cladding. In some applications, additive manufacturing is being used, especially where the complicated geometry of the part results in extremely expensive and time-consuming machining or low-volume production [1,2,3,4,5]. All of the laser-based AM techniques use a focused energy source for building parts layer by layer, which results in very specific solidification conditions and sometimes leads to obtaining nonequilibrium structures. Those techniques force us (or maybe allow us) to redefine the design and manufacturing schemes and verify knowledge about the properties of even the most basic and well-known materials that have been used successfully for years until now. The properties of additively manufactured steels have been under extremally intensive investigation in recent years worldwide in terms of microstructure, mechanical properties, and corrosion resistance [6,7,8,9,10,11,12,13,14,15,16,17,18,19,20]. When extremely fast cooling and layer-by-layer forming are applied, the properties of materials are very different from those that we are used to and that result from casting followed by hot or cold work, as is usually the case in the industry. The problems related to these specific cooling conditions are known since they are similar to those that we face during the welding of steel [3,21,22]. However, in the case of additive manufacturing, the cyclic heat treatment and several remelting of each layer make the case even more complicated. Recently, it was shown that the corrosion resistance of LENS^®^ manufactured samples might be better than that of the wrought alloy [23], and the mechanism was also described [24]. It has also already been reported [25] that each grain in AM 316L had a net structure composed of subgrains and formed a so-called net structure due to very fast dendritic crystallisation [26]. Saeidi et al. [27] further found Mo enrichment and a high concentration of dislocations at subgrain boundaries. This was attributed to the fast cooling rates and high gradients of the temperature during the AM process. This unique microstructure had a significant influence on the mechanical properties. For example, quite recently, Wang et al. showed that SS316L fabricated by SLM had an ultimate tensile strength of ~600 MPa and a total elongation of 70%, which was significantly greater than that of wrought SS316L [28]. Therefore, the extraordinary combination of strength and ductility that offers promising prospects for AM SS316L application can be reached for some combination of manufacturing parameters. Direct energy deposition methods usually have quite broad processing windows that can be applied for the manufacturing of samples, but sometimes a slight change may result in obtaining a structure with residual ferrite, which is already described [29]. In the case of other steels that were additively manufactured, the change in cooling rate related to the thickness of the deposited layer may greatly influence the phase composition and, thus, the properties [30]. Depending on the type of material deposited, it can have significantly different properties, but the deposition efficiency may vary by almost an order of magnitude, causing the process to be economically effective or not [31].

In this study, we investigate the influence of layer thickness and deposition rate on the microstructure, mechanical properties and corrosion resistance of 316L stainless steel manufactured by the LENS^®^ method. The results show that significantly different processing parameters may lead to very similar results in terms of mechanical properties and phase composition, but the corrosion resistance properties may differ between samples.

## 2. Materials and Methods

**Figure 1:**
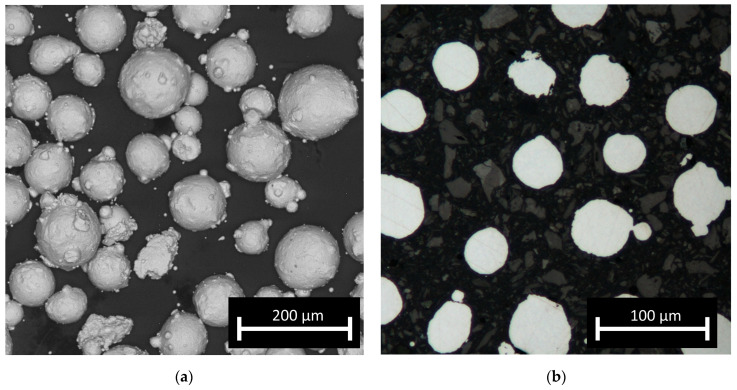
Micrographs of feedstock powder: (**a**) optical microscopy image of particles and (**b**) SEM image of powder showing its morphology.

The feedstock material used to manufacture the test samples was AISI 316L steel powder (TLS, Germany). The as-received gas (argon) atomised powder was additionally sieved in a vibrational sieving station for 50 min to obtain a fraction from 44 to 106 μm. The post-sieving particle size analysis (IPS-U, Kamika, Poland) showed that approximately 97% of the particles were within the desired particle range; the particles were almost perfectly spherical (Figure 1a). For the macroscopic examination, samples were ground, polished, etched (swabbing by ASTM E407 Kallings Etchant) and observed using metallographic microscopy (KEYENCE VHX-950F). To examine the microstructures, all samples were observed using optical microscopy (Nikon Eclipse MA200) as well as SEM (FEI, QUANTA 3D) in SE mode. Observations by optical microscopy of the cross-sections of the particles (fixed in thermoset resin) revealed no internal porosity in the powder (Figure 1b) and no significant amount of so-called satellites, which should not influence the powder flowability. The chemical composition and particle shape were confirmed by scanning electron microscopy (SEM) (Quanta 3G FEM Dual Beam, FEI) observation and EDS analysis and corresponded well with the nominal composition of 316L steel. The amount of carbon was not evaluated quantitatively due to the measurement limitations of energy-dispersive X-ray spectroscopy (EDS) for light elements. The EBSD analysis was carried out at a voltage of 20 kV. Data were collected at two magnifications of 200 and 2000 times in steps of 3 and 0.3 micrometers, respectively. Grain boundary analysis was performed assuming two angular ranges from 2 to 15 degrees and above 15 degrees. The STEM images were taken with the use of a Hitachi HD2700 microscope, with 200 kV acceleration voltage and a HAADF detector. The phase composition of the obtained samples was investigated based on X-ray diffraction spectroscopy (XRD) patterns collected with the use of an Ultima IV (Rigaku, Japan) diffractometer with a cobalt anode (Kα1 λ = 1.788965 Å), parallel beam geometry and a linear DeteX Ultra counter. A cobalt anode was used to avoid fluorescence and to obtain low noise measurements. Parallel beam geometry also reduces the Kβ radiation contribution to less than 1% of the intensity.

Corrosion performance was measured by two independent methods. The first one relied on the determination of the electrochemical corrosion parameters (general and pitting). The corrosion process consisted of placing the examined samples in an electrochemical cell with 0.9% NaCl. The examined samples served as the working electrodes (WE), while the Pt electrode and Ag|AgCl electrode were used as counter (CE) and reference (RE) electrodes, respectively. In order to ensure constant and repetitive exposed surface area of the samples to the electrode, an O-ring seal with a 14 mm diameter was applied. All the electrochemical experiments were performed using Atlas-Sollich 0531 potentiostat. In order to obtain the polarisation curves, firstly, open cell circuit potential (OCP) was measured for 2 h, and immediately after, 100 mV below the last value of the OCP polarisation experiment was started with potential increasing with 1 mV/s rate.

The second method of the corrosion performance examination was based on a neutral salt spray test (NSST). The samples were examined in an inert salt spray atmosphere according to PN-EN ISO 9227:2012 after 336 h. Before testing, the samples were kept for 24 h at 23 ± 2 °C and relative humidity 50 ± 5%. The conditions of the experiment are shown in Table 1.

Hardness tests were performed using the universal (Vickers/Brinnel) testing machine HPO 250. The indentation force of 98.07 N (HV10) was used for 10s for each indentation. Microhardness distribution was measured using Shimadzu (type M) tester with 0.098 N (HV0.01) force and 10 s for each indentation. Tensile properties were examined for a total of twelve samples (three samples for each material state and the reference material). The samples were cylindrical, and the diameter of the measurement section was 4 mm. A strain gauge (Instron) with a measuring 12.5 mm measurement range was used. Instron 8501 tensile testing machine was used in the quasi-static deformation tensile test speed range. The samples were cut in parallel to the substrate by EDM as cylinders and machined with the use of a CNC lathe in order to obtain the final sample shape.

## 3. Experimental Conditions and Procedure

Test samples were manufactured with the LENS MR-7 (Optomec, Albuquerque, NM, USA) system. The machine was custom-made and equipped with 4 powder feeders, a melt pool control system and thermal imaging of the molten metal pool. The maximum working envelope of the machine was 300 × 300 × 300 mm. During the manufacturing process, a continuous feed of argon gas (5N purity) of 4 L/min was used to feed the powder, and 20 L/min for protection of the laser optics was blown through the centre nozzle. Each sample was built as a cuboid with dimensions of 50 mm × 75 mm × 10 mm (X, Y, Z). The manufacturing parameters are given in Table 2. The parameters were chosen to change the layer thickness significantly while keeping the laser power and powder feed rate at reasonably constant levels. Since the samples of high XY- and low Z-dimensions were built, high deformation of samples due to thermal stress was observed, which led to substrate deformation. For that reason, after deposition, the samples were stress relieved, annealed at 400 °C for 5 h and cooled in the furnace. Before the annealing process, slices of material from each sample were cut to analyse and compare the microstructure and hardness of the manufactured parts before and after heat treatment.

## 4. Results and Discussion

### 4.1. Macroscopic and Microscopic Observations

**Figure 2:**
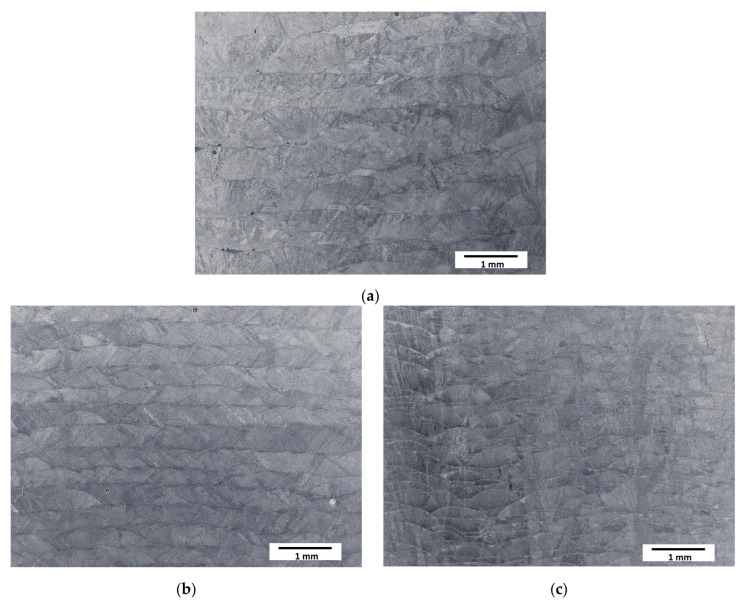
Optical microscopy micrographs of manufactured samples: (**a**) Sample A (0.7 mm layer thickness); (**b**) Sample B (0.4 mm layer thickness); (**c**) Sample C (0.2 mm layer thickness).

The typical paths (clads) resulting from the deposition of subsequent layers during the LENS^®^ process were observed at low magnifications (Figure 2). The structure of the samples was coherent—no cracks or a significant amount of pores were observed. The layers were found to be, as assumed, thicker for slower laser feed rates and thinner for faster feed rates. 

**Figure 3:**
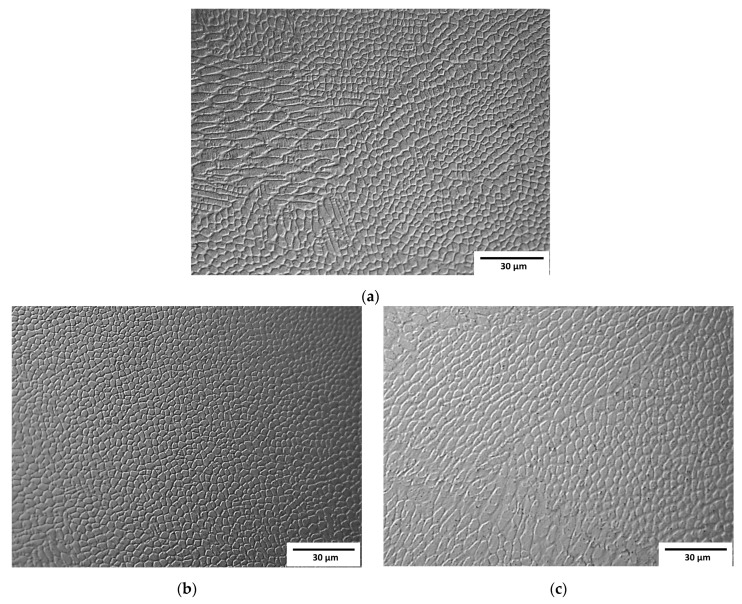
Micrographs of samples observed by optical microscopy: (**a**) Sample A (0.7 mm layer thickness); (**b**) Sample B (0.4 mm layer thickness); (**c**) Sample C (0.2 mm layer thickness).

**Figure 4:**
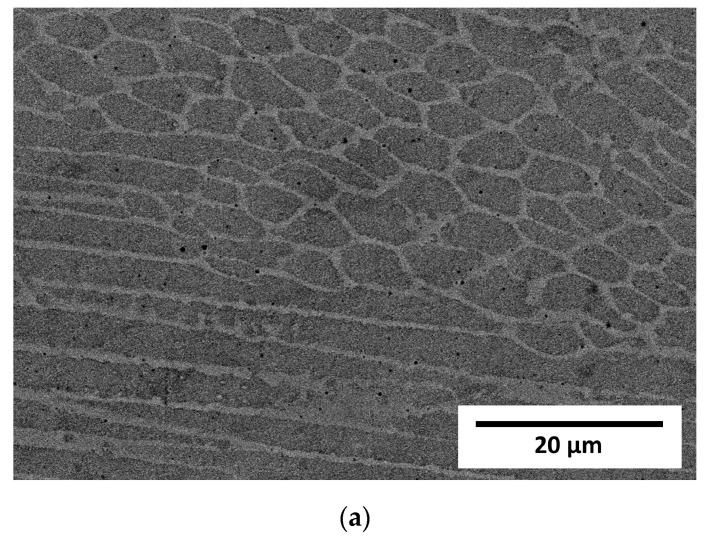

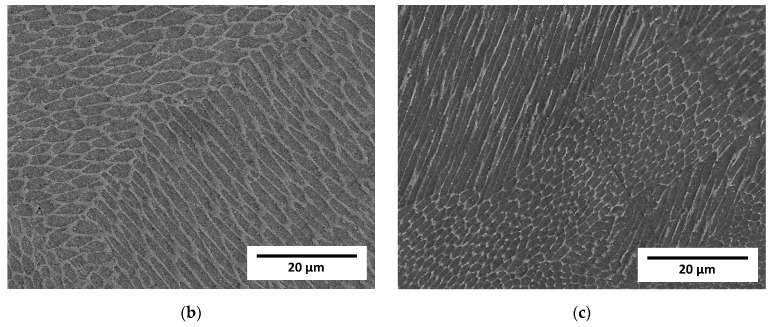
Micrographs of LENS^®^ manufactured samples taken on a scanning microscope in SE mode. (**a**) Sample A (0.7 mm layer thickness); (**b**) Sample B (0.4 mm layer thickness); (**c**) Sample C (0.2 mm layer thickness).

Observation at higher magnifications allowed the observation of a typical cellular structure (Figure 3) resulting from the rapid dendritic solidification, which is discussed in the following sections. There is an obvious increase in the size of single “welds” with decreasing laser feed rate, but any obvious difference in the size of the cells was a subjective observation. Scanning electron microscopy of the etched samples (Figure 4) showed images very similar to the optical ones, i.e., cellular structures were observed. Additionally, black spots were observed, suggesting some kind of porosity or inclusions. Keeping in mind that these were SE observations, the colour did not represent the chemical composition, and EDS point chemical composition measurements were performed, which showed high silicon and oxygen contents in the measured areas; this suggested that those were likely remnants from the metallographic preparation (colloidal silica likely penetrated the pores).

Visible differences between the cells and dendritic regions were likely caused by etching [32] and possibly chemical segregation, which was quite obvious and reported previously [24,29].

### 4.2. Phase Analysis

**Figure 5:**
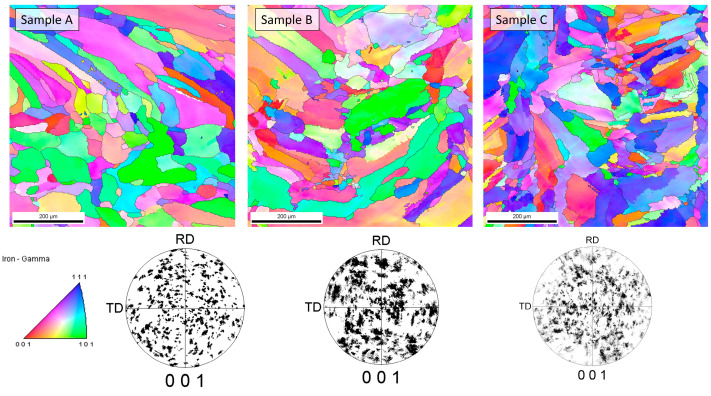
Results of EBSD analysis of samples.

The cellular structure described above was very difficult to describe quantitively due to large differences in apparent cell size caused by the direction of the metallographic cross-sections. For that reason, the cell size analysis was not performed because, with such high uncertainty of the results, the apparent trend might be more correlated with the chosen observation area than with the processing parameters. Additionally, it must be remembered that the cells that can be clearly seen using the optical microscope were not grains according to the typical definition of the grain, and only cross-sections of the dendrites formed during rapid solidification. For that reason, electron backscatter diffraction (EBSD) analysis was performed to reveal the real grain structure, where grains were defined as areas divided by high-angle grain boundaries. EBSD analysis was also performed to identify phases other than the austenite phase (especially ferrite and possibly sigma) as well as to measure the real grain size. Observations were taken at two magnifications, 200× and 2000×, and the first ones are shown in Figure 5. Crystallographic orientation maps and pole figures suggest that the grains were oriented randomly, and thus no texture was visible in the observed areas. This was quite an unusual finding in consideration of the nature of the additive manufacturing process, which very often results in the preferred orientation of the grains due to the specific solidification conditions. Phase analysis performed with EBSD showed no occurrence of the alpha phase (ferrite). Exemplary results from the EBSD observation are given in Figure 5. Again, due to the very elongated grains and uniaxial grains that are visible in the picture, as well as relatively bad statistics even at such low magnification (large grains), the quantitative analysis was considered to be unreasonable. In contrast to the EBSD results for small areas, the results obtained from XRD phase analysis showed significant texture, which could be judged from the difference in the relative intensities of the obtained XRD patterns compared to the reference PDF card. This observation further supports the finding that the preferred orientation and texture were strongly dependent on the scale at which the sample is observed. Moreover, in the case of additive manufacturing, more than in the case of any other manufacturing techniques, the result of the observation was extremely dependent on the area that was observed as well as the direction in which the sample was cut, especially in bulk (not thin-walled) structures. For that reason, it was finally decided not to calculate the grain size, even after the EBSD examination. At the microscale, a randomly chosen area may be within a single clad of unknown direction, and thus, its average crystal orientation is likely not to be representative of the total volume of the material. Therefore, the local crystallographic orientations may be interesting to some extent from the informational point of view; however, they do not influence the macroscopic properties of the material. The most important information obtained from EBSD was that no ferrite was observed in the samples. Due to the specific cooling conditions, despite the chemical composition (high nickel content), in the case of additively manufactured samples, it is possible that delta ferrite exists due to local deviation of the chemical composition as well as extremely fast cooling [29].

**Figure 6:**
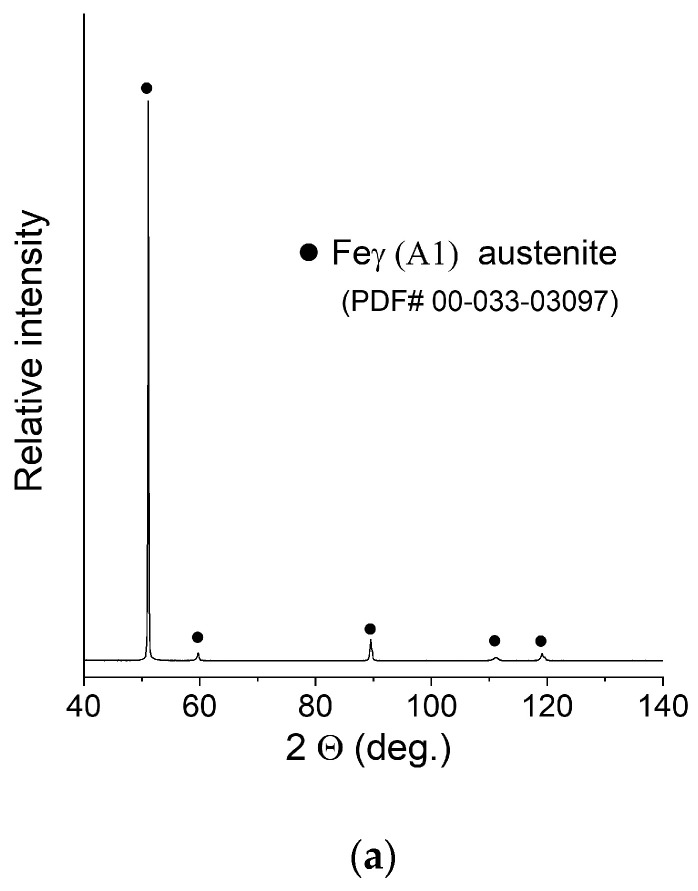

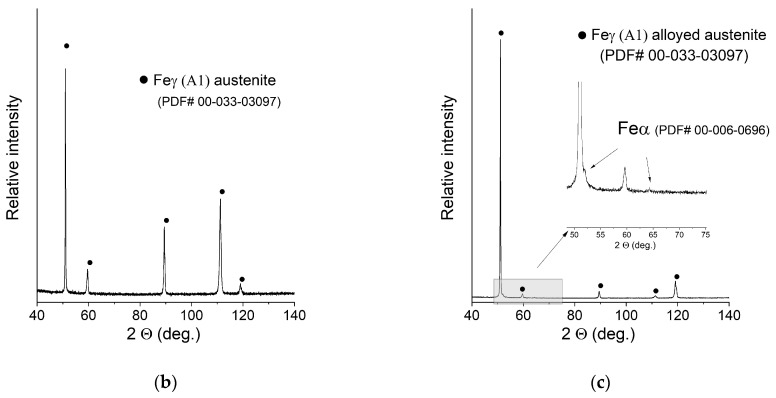
XRD patterns of the obtained samples: (**a**) Sample A (0.7 mm layer thickness); (**b**) Sample B (0.4 mm layer thickness); (**c**) Sample C (0.2 mm layer thickness).

XRD patterns of the manufactured samples can be seen in Figure 6. The measurement was taken with the use of a bulk material sample without pulverising the sample to maintain the phase composition (avoid formation of the deformation martensite). Due to this fact, significant texture effects were expected, and big differences in the relative intensities of the peaks were observed. After careful examination, extremely weak peaks assigned to the presence of ferrite were found in the XRD pattern of sample C (0.2 mm layer thickness). 

No NiCr sigma phase was found to be present. Due to the weak intensity of the ferrite peaks, no quantitative analysis was performed. In order to confirm or deny the presence of the ferrite in a measurable amount, the ferrometer was used. The results shown in Table 3 support the initial observations and prove that an almost pure austenitic structure was obtained in all cases.

### 4.3. Hardness Measurement and Distribution of Microhardness

**Figure 7:**
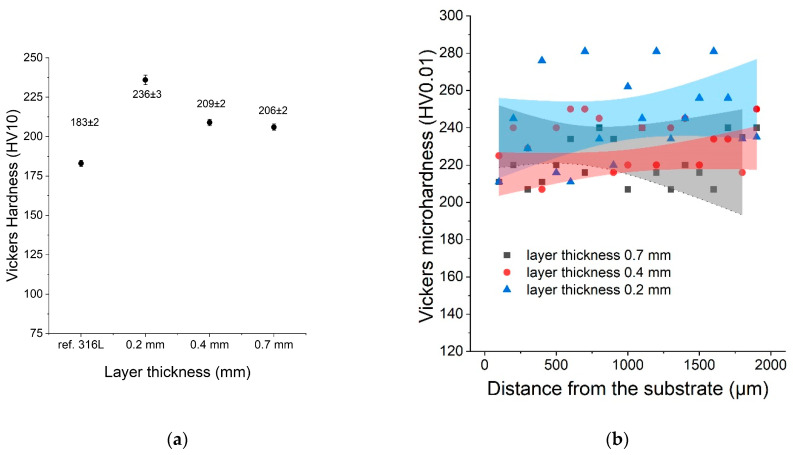
(**a**) Hardness as a function of the layer thickness compared with the reference sample; (**b**) microhardness distribution as a function of distance from the substrate.

The hardness of all samples was measured using the Vickers method (HV10). This study was conducted to compare the hardness of the LENS^®^ sample with the hardness of the reference 316L sample. The average hardness was measured (HV10) for each sample as well as the microhardness distribution as a function of distance from the substrate. After analysing the results, it was found that the hardness of the LENS^®^ samples was significantly greater than the hardness of the reference sample despite their postprocessing annealing at 500 °C. Sample C (0.2 mm) was characterised by the greatest hardness, likely because it was manufactured with the fastest deposition velocity (20 mm/s). The results of both hardness and microhardness measurements are shown in Figure 7.

The microhardness showed significant variations, and that was likely caused by inhomogeneities in each sample (sometimes 200–280 HV) at the microscale.

### 4.4. Tensile Properties

**Figure 8:**
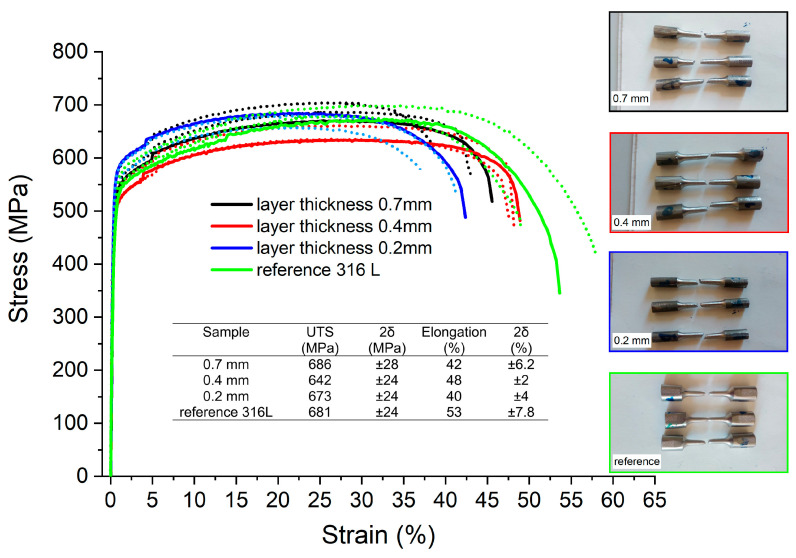
Tensile test results and stress–strain curves obtained for additively manufactured samples.

The results of the tensile test are shown in Figure 8. After calculating the average ultimate tensile strength (UTS) and yield strength (YS) values and estimating the uncertainties (measured as double standard deviation 2δ), it was concluded that there were no statistically meaningful differences between the tensile properties of both additively manufactured and reference samples. The only measurable difference was seen between the elongation observed for the reference sample (53 ± 7.8%) and the sample with the smallest layer thickness (40 ± 4%).

### 4.5. Corrosion Resistance

#### 4.5.1. Electrochemical Corrosion

**Figure 9:**
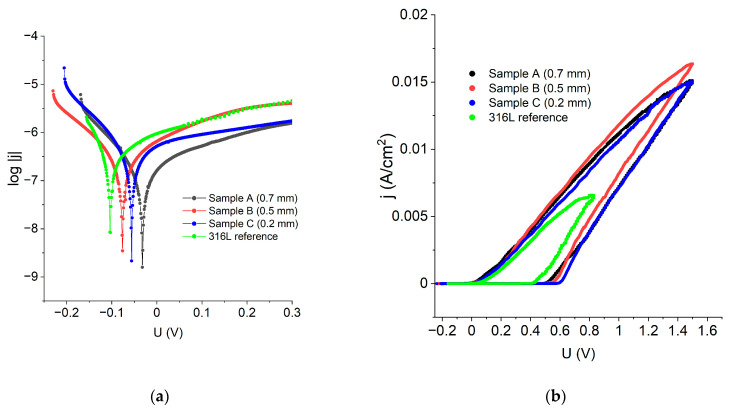
(**a**) polarization curves, in log|j|vs. E coordinates of the samples obtained via additive manufacturing and a reference 316L sample; (**b**) polarisation curves of the samples obtained via additive manufacturing and a reference 316L sample.

In order to examine the corrosion performance of the manufactured materials, the experimental results were represented in log|j| vs. E coordinates to make Tafel’s law extrapolation possible. Figure 9a reveals that all the LENS^®^ manufactured samples have greater corrosion potential than the standard 316L steel, which means that the LENS^®^ manufactured samples are less prone to corrosion than the reference 316L steel. Data obtained from the Tafel extrapolation are gathered in Table 4 and confirm these findings; when compared to the reference, the LENS^®^ manufactured samples had even corrosion potential as high as −0.03 V vs. Ag|AgCl when compared to the 316L steel (−0.10 V vs. Ag|AgCl). Corrosion current density was found to be comparable among the examined samples; however, the lowest values, translating into the lower corrosion rates, were also achieved for the LENS^®^-manufactured sample (Table 4).

Figure 9b shows polarization curves in j vs. E coordinates that allowed for estimating such quantities as pitting/breakthrough potential (E_b_), repassivation potential (E_cp_) and width of the hysteresis loop. All the calculated corrosion performance parameters are shown in Table 4.

The data analysis revealed that the best corrosion performance has sample A because it has the highest corrosion potential and the lowest corrosion current density and corrosion rate. This analysis showed that the LENS^®^ samples had better general corrosion resistance than the reference 316L steel sample. There was no tendency for the corrosion resistance to increase or decrease with increasing feed rate and layer thickness; i.e., there was a lack of a clear relationship between the parameters of the LENS^®^ samples and resistance to general corrosion.

In the case of the resistance towards pitting corrosion, a decrease in corrosion resistance with increasing feed rate and a reduction in layer thickness was observed. A sample with a layer thickness of 0.2 mm was found to possess the lowest resistance to this type of corrosion. The sample that was the most resistant to the pitting corrosion was a sample manufactured with a 0.7 mm layer thickness, which was indicated by the width of the hysteresis—this is the sample that recovers the most rapidly the passive protective layer on the surface. However, in this case, the LENS^®^ sample possesses much lower resistance to pitting corrosion than the reference sample, wherein the hysteresis width was only 0.43 V. The obtained results are in very good agreement with the results shown for another experiment by Nie et al. [24] and Revilla et al. [32].

#### 4.5.2. Corrosion in Salt Chamber

Further corrosion tests in a salt chamber were conducted using three samples of each additively manufactured sample and two reference samples. The experiment lasted 336 h. Assessment of corrosion was based on visual analysis of surface samples tested according to PN-EN ISO 1289: 2002 standard, as follows:

Protection factor Rp—resistance to corrosion of the substrate—as:

10—there is no corrosion of the substrate/no defect;
9—corrosion of the substrate < 0.1% of the sample substrate;
8—corrosion of the substrate > 0.1%, <0.25% of the sample substrate;
7—corrosion of the substrate > 0.25% and <0.5% of the sample substrate;
6—corrosion of the substrate > 0.5%, <1.0% of the sample substrate;
5—corrosion of the substrate > 1.0% and <2.5% of the sample substrate;
4—corrosion of the substrate > 2.5% and <5.0% of the sample substrate;
3—corrosion of the substrate > 5.0% and <10% of the sample substrate;
2—corrosion of the substrate > 10%, <25% of the sample substrate;
1—corrosion of the substrate > 25%, <50% of the sample substrate;
0—corrosion of the substrate > 50% of the sample substrate.

The results of corrosion based on the salt chamber test are given in Table 5. The analysis showed that the corrosion resistance decreased with increasing feed rate and decreasing layer thickness for the additively manufactured samples. More interestingly, the samples manufactured with 0.7 and 0.4 mm layer thicknesses were found to possess better corrosion resistance than the reference sample.

The neutral salt spray test revealed that after the 336 h long exposure to the aggressive, chloride-rich environment, 0.4 mm and 0.7 mm samples revealed better corrosion performance than the standard. These results are in concordance with the polarisation experiments, especially with the corrosion data acquired from the Tafel plots (Figure 9a).

### 4.6. Microstructure Type and Solidification Mechanisms

The microstructure of 316L steel samples manufactured by LENS^®^ technology resembled a net, ichthyosis or snakeskin. The microstructure of austenitic stainless steel is highly dependent on its chemical composition.

Phase changes during the crystallisation of such steels can be run according to four different types (based on a simplified ternary phase diagram of stainless steel—Fe-Cr-Ni). Each of these methods is characterised by different microstructures depending on the ratio CrE/NiE:

1. type A L → L + γ → γ for CrE/NiE < 1.25;
2. type AF L → L + γ → L + δ + γ→ γ + δ→ γ for <1.25 CrE/NiE < 1.48;
3. type FA L → L + δ → L + δ + γ→ γ + δ→ γ for <1.48 CrE/NiE < 1.95;
4. type F L → L + δ → δ → δ + γ→ γ for CrE/NiE > 1.95.

where:

CrE—equivalent of chromium in stainless steels (%);
CrE = Cr % + Mo % + 1,5 Si % + 0.5 Nb %;
NiE—equivalent of nickel in stainless steel (%);
NiE = Ni % + 30 (C+ N)% + 0.5 Mn %.

Steels with austenitic structure transformation from the FA to AF type (transformation of primary delta ferrite dendrites to dendrites of austenite) are conditioned by the cooling rate because, along with its growth, during crystallisation, the tendency to precipitate metastable austenite increases due to the high initial rate of heat removal. The formation of metastable austenite from liquid metal becomes possible when supercooling is greater than the difference between the temperature of the liquidus balance of delta ferrite and the temperature T0 of this metastable γ (critical value of supercooling). This relationship is represented by the equation $∆T>∆T_{0,\gamma }=T_{1,\delta }-T_{0,\gamma }$. When supercooling exceeds $∆T_{0,\gamma },\;$the terms of the kinetic phase transition are determined either by the balance of the delta ferrite phase or the phase of the metastable austenite. This is because there is still possible crystallisation of primary ferrite. The formation of metastable austenite as the primary phase is thus conditioned by the increase in supercooling. Growth of the austenite phase is first initiated in the supercooling place of steel, provided that the value of critical supercooling is reached. Accordingly, the δ-ferrite phase can increase in the alloy gradually in the form of cells or dendrites when the supercooling is below the critical value. However, as soon as supercooling reaches a critical value, the metastable austenite phase becomes the only main and fundamental phase in the alloy. Another important transformation during the crystallisation of austenitic steels is the transformation from dendrites γ to cells γ. This transformation is activated when there is an even stronger increase in the cooling rate. Such transformations are possible through the disintegration of the lateral branches of dendrites due to the increase in the temperature gradient and speed of grain growth during column crystallisation. When the growth rate reaches a critical value, the arms of the dendrites disintegrate, and the crystallisation of the cells becomes the main form of growth in the specimen.

For the manufactured LENS^®^ samples and the reference sample, to determine the original model of crystallisation, chromium and nickel equivalents were calculated, and the CrE/NiE ratios were determined. This was performed after noticing the measurable differences in the chemical composition of the samples, although they were manufactured from the same batch of powder. It is very likely that the slower feed rate caused both chromium and nickel depletion but was still in the range proper for 316 L SS. Calculations based on measured chemical composition showed that all the samples crystallised according to the AF model in a dendritic form of austenite formed on a net of primary ferrite (Table 6). In the LENS^®^ process, a very fast cooling rate caused the resulting microstructure to be dendritic with a large proportion of cell grains.

**Figure 10:**
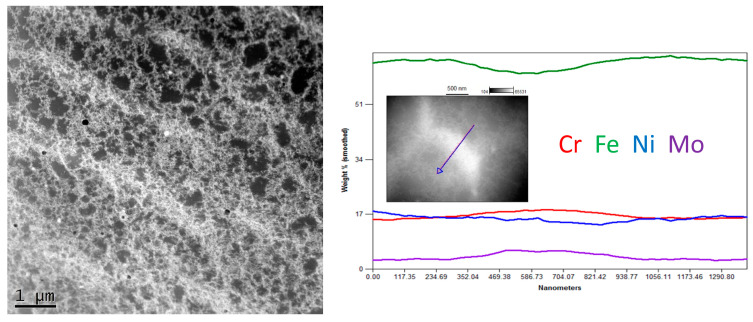
Exemplary STEM image of Sample C (0.2 mm) with linear chemical composition analysis.

The STEM observation together with EDS line scan analyses showed quite a noticeable chemical segregation at grain boundaries, as in Figure 10. After a thorough analysis of all the results of the research, it was concluded that the difference in the colour of the grain boundaries resulted from a greater density of dislocations at the grain boundaries than in the grains. Another reason for this difference may have been the same etching process, which operated more strongly on the borders than the grains. It was therefore concluded that the characteristic microstructure in the form of a net or ichthyosis was a purely austenitic microstructure with a dendritic and cellular morphology formed on the original net of delta ferrite, with a greater density of dislocations distributed on the grain boundaries. Significant segregation of molybdenum is also noticeable. As already discussed [24,32], the three-dimensional network with different chemical segregations and significantly different dislocations density causes the increase in stability of the protective film formed during passivation, thus increasing the corrosion resistance. 

## 5. Conclusions

After a comprehensive analysis of the test results, the following conclusions were drawn:

In the chosen processing window (6–20 mm/s, 0.7–0.2 mm layer thickness), which was chosen in order to produce the highest differences in crystallisation rates, samples produced by the LENS^®^ method possess a purely austenitic structure with no measurable amounts of delta ferrite despite local variations in chemical composition caused by fast cooling. Each of the manufactured samples possessed a cellular structure formed due to rapid solidification. The cells were found to show chemical composition segregation, but based on the STEM observation and literature analysis, the density of the dislocations is also likely different in the cells as compared to interdendritic regions. 

The hardness of LENS samples increases slightly with increasing feed rates and decreasing thickness of layers. The microhardness distribution suggests microscale inhomogeneities in chemical composition.

The tensile properties of the additively manufactured samples were statistically the same as those of the reference sample. The elongation of the sample deposited with 0.2 mm layer thickness was noticeably worse than that of the reference sample.

The corrosion performance of the LENS^®^ manufactured samples revealed their better corrosion resistance than the reference sample, but a relationship between the production parameters and corrosion resistance was not found for the general corrosion due to very small differences. However, a relationship was found for pitting corrosion resistance, i.e., this resistance decreases with increasing feed rate and decreasing layer thickness; nevertheless, much further mechanistic study and understanding is required. The results from observing the samples after neutral salt spray tests showed relationships between production parameters and corrosion resistance, i.e., resistance decreases with increasing feed rate and decreasing layer thickness.

## Figures and Tables

**Table 1:** Parameters of the salt chamber experiment.

Parameter	Value
Concentration of NaCl solution	(50 ± 5) g/dm^3^
Working temperature of salt chamber	34.0–35.2 °C
pH of 5% NaCl solution	6.20–6.85
pH of the condensate	6.55–6.90
Density of the condensate	1.036 g/cm^3^

**Table 2:** Manufacturing parameters of the samples.

Sample	Laser Feed Rate [mm/s]	Layer Thickness [mm]	Powder Feed Rate [g/min]	Laser Power [W]	Hatch [mm]	Laser Spot Size [mm]
A	6	0.70	7.85 ± 0.1	435	0.75	0.8 ± 0.1
B	10	0.40	7.85 ± 0.1	450	0.70	0.8 ± 0.1
C	20	0.20	7.85 ± 0.1	450	0.60	0.8 ± 0.1

**Table 3:** Ferrite content in the samples measured by ferrometer.

Sample	Ferrite (%)
A (0.7 mm)	0.07 ± 0.04
B (0.4 mm)	0.1 ± 0.04
C (0.2 mm)	0.07 ± 0.04
reference 316 L	0.07 ± 0.04

**Table 4:** List of parameters of electrochemical corrosion (pitting and general) for all analysed samples.

Sample	E_corr_ [V]	j_corr_ [A/cm^2^]	V_corr_ [g/cm^2^h]	E_b_ [V]	E_cp_ [V]	Hysteresis [V]
LT 0.7 mm	−0.03	2.21 × 10^−8^	6.41 × 10^−8^	0.48	−0.06	0.54
LT 0.4 mm	−0.08	4.44 × 10^−8^	1.29 × 10^−7^	0.48	−0.08	0.56
LT 0.2 mm	−0.05	3.57 × 10^−8^	1.00 × 10^−7^	0.57	−0.07	0.64
reference 316L	−0.10	3.49 × 10^−8^	1.01 × 10^−7^	0.38	−0.05	0.43

**Table 5:** The results for corrosion resistance after testing in the salt chamber.

Sample	The Appearance of Samples after the Tests of Corrosion in an Inert Salt Spray Chamber	
	Initial State	After 336 h in the Chamber
0.7 mm	R_p_ = 10.0	R_p_ = 9.7
0.4 mm	R_p_ = 10.0	R_p_ = 9.3
0.2 mm	R_p_ = 10.0	R_p_ = 8.7
316L reference	R_p_ = 10.0	R_p_ = 9.0

**Table 6:** Chromium and nickel equivalents, the coefficient of CrE/NiE, and the type of crystallisation.

Sample	CrE [%]	NiE [%]	CrE/NiE	Crystallisation Type
Sample A LT 0.7 mm	18.32	13.01	1.41	AF
Sample B LT 0.4 mm	19.30	13.86	1.39	
Sample C LT 0.2 mm	19.37	13.97	1.39	
316 L—reference	19.26	13.70	1.41	

## Data Availability

Data are available upon request.
